# Quasi–two-dimensional ferroelectricity with multiple switchable polarization states in N-H coinjected perovskite manganites

**DOI:** 10.1126/sciadv.adx3747

**Published:** 2025-10-01

**Authors:** Xian-Kui Wei, Feng Liu, Yi Wang, Zhiyao Liang, Pengpeng Liu, Ying Zhou, Lei Cao, Pengfei Cao, Yi Li, Slawomir Prucnal, Oleg Petracic, Yinguo Xiao, Penghan Lu, Ivan Lazić, Shuai Dong, Shengqiang Zhou, Rafal E. Dunin-Borkowski

**Affiliations:** ^1^ College of Chemistry and Chemical Engineering, Xiamen University, Xiamen 361005, China.; ^2^ Ernst Ruska-Centre for Microscopy and Spectroscopy with Electrons, Forschungszentrum Jülich GmbH, 52425 Jülich, Germany.; ^3^ Innovation Laboratory for Sciences and Technologies of Energy Materials of Fujian Province (IKKEM), Xiamen 361005, China.; ^4^ Key Laboratory of Quantum Materials and Devices of Ministry of Education, School of Physics, Southeast University, Nanjing 211189, China.; ^5^ School of Advanced Materials, Peking University, Shenzhen Graduate School, Shenzhen 518055, China.; ^6^ Institute of Ion Beam Physics and Materials Research, Helmholtz-Zentrum Dresden-Rossendorf, Dresden 01328, Germany.; ^7^ Jülich Centre for Neutron Science (JCNS-2), JARA-FIT, Forschungszentrum Jülich GmbH, Jülich 52425, Germany.; ^8^ Materials and Structural Analysis Division, Thermo Fisher Scientific, Eindhoven, Netherlands.

## Abstract

Topotactic transformation such as hydrogenation serves as a powerful approach to engineering material functionality. However, challenged by direct imaging of light elements and clarifying their role, dual ion-based topotactic tranformation has been poorly explored so far. Here, we report on NH_3_ plasma–induced ferrodistortive phase transition in N*_x_*H*_y_*(La,Sr)MnO_3-δ_ films (0 < *x* < 0.2, 0.5 < *y* < 1.0, and δ ≈ 0.125, 0.25, and 0.5), where the injected H and N are resolved to enhance the polar order along with antisite defects by atomic-resolution electron microscopy. Besides unveiling the mediation of structural modulation and N-H competition by oxygen-vacancy ordering degree, our piezoresponse force microscopy unravels a unique quasi–two-dimensional (q2D) ferroelectricity in fourfold modulated brownmillerite phase (δ ≈ 0.25), which offers a series of switchable polarization states by an applied electric field. Unlike all-known ferroelectrics, the q2D ferroelectrics establishes a promising material platform for design of future electronic devices such as multistate information storage.

## Teaser

The NH_3_-based topochemistry in manganites leads to quasi-2D ferroelectricity with multiswitchable states for information storage.

## INTRODUCTION

Controllable material design via topotactic transformation provides great opportunity and flexibility for exploiting functional materials and device application, e.g., information storage, energy catalysis, wearable electronics, etc. (*1*). Without disrupting the overall crystal framework, the topotactic transformation allows for precise control over chemical and structural changes, which gives rise to enhanced and even previously unidentified functionalities, e.g., the electrochromatic effect, optical modulation, proton conductivity, and superconductivity in H*_x_*SrCoO_3_, H*_x_*VO_2_, and H*_x_*(Nd,Sr)NiO_2_ (*2*–*5*), respectively. Apart from hydrogenation, anionic exchange exemplified by substitution of oxygen by nitrogen, which enables alteration of oxidation state and coordination environment, has also been widely studied both in theory and experiment for pursuit of highly efficient solar energy conversion and robust ferroelectric (FE)–based sensors (*6*–*9*), e.g., in InSeO_2_N and LaWN_3_. Nevertheless, as for the codoping of H and N species, their role in retrofitting structure and property of oxides has rarely been explored so far.

Despite serving as a powerful tool to engineer material structure and property, factors such as temperature, atmospheric pressure, and reaction pathway (*10*) that may affect the topotactic transformations are many, and the subject-guest interaction is far from being elaborated. Taking the N-to-O exchange as an example, where the ammonia (NH_3_) is commonly used as the reagent source of nitrogen, although the electroneutrality law suggests that the N substitution amount should be increased by proton injection, the replaceable concentration in perovskites is usually quite low, e.g., in SrVO_2.7_N_0.2_ and LaTiO_2.7_N_0.3_ (*7*, *9*). Following this unsolved mystery, one key question is that the nitrogen is naturally assumed to replace oxygen. However, a direct experimental evidence is rare so far. On the other hand, during the ammonia treatment, the hydrogen can easily intercalate into the oxide lattices. Nonetheless, its role in modifying the material structure is largely ignored in previous studies. These remnant issues leave a great blank for engineering functionality of oxides via the N-H coinjection.

To address the above issues, the structure and electrical property evolution of NH_3_ plasma–treated (La_0.7_Sr_0.3_)MnO_3-δ_ (LSMO) thin films are investigated by controlling the concentration of oxygen vacancies (Vos). By combining different microscopy and spectroscopy techniques, our study reveals a competitive interplay between Vo order degree and N-H coinjection, where the H and N species are resolved at different interstitial and cation-oxygen dual sites, respectively. Stimulated by atomic antisite defects, long-range ferrodistortive (FD) orders are prevailingly observed in structural phases with different Vo arrangements. Specifically, we found that the Vo ordering–induced fourfold modulated phase (δ ≈ 0.25) can be classified as a quasi–two-dimensional (q2D) FE. Besides a strong anisotropy in resistivity, it offers a series of switchable polarization states by an applied electric field. Our findings shed light on design of functional materials and devices, e.g., multistate information storage, via NH_3_-based topotactic transformation.

## RESULTS

### Structure and resistivity modulation

**Fig. 1.**
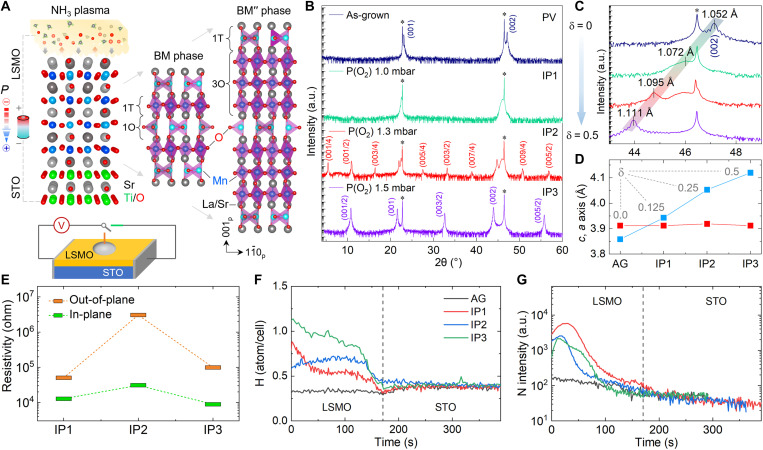
N-H codoping induced structural and resistivity modulation. (**A**) Schematic NH_3_ plasma treatment, its potential poling role via dynamic anionic exchange at the plasma/oxide interface, and structural evolution from the PVL phase to two- and fourfold modulated BM and BM” phases. (**B**) X-ray θ−2θ diffraction patterns of AG (*P*_O2_ = 1.5 mbar) and IP1, IP2, and IP3 NH-LSMO films initially grown under *P*_O2_ = 1.0, 1.3, and 1.5 mbar, respectively. The star symbols denote the diffraction peaks of the STO substrates. a.u., arbitrary units. (**C** and **D**) Magnified view of (002)_p_ reflections and corresponding lattice parameters, respectively. (**E**) In-plane and out-of-plane resistivity of the NH-LSMO samples measured at room temperature on the basis of the circuit illustrated above. (**F** and **G**) SIMS profiles of H and N measured as a function of sputtering time for the AG and NH-LSMO films, respectively.

When the LSMO films are subject to the NH_3_ plasma environment, one can foresee that there exists a prominent chemical potential difference at the plasma-oxide interface. Because of the thermodynamic activity, different chemical species start to interact with each other near the interface, e.g., the N^3−^-to-O^2−^ anion exchange and combination of protons with lattice oxygen into H_2_O molecules (Fig. 1A). Before reaching an equilibrium state, the chemical potential difference may act as an “electric field” to continuously pole the sample. As a result, the polar order tends to emerge from the surface and then penetrate into the oxide. Similar to high-temperature vacuum annealing (*11*), the perovskite-like (PVL) to brownmillerite (BM) phase transition can be expected in this process, which is accompanied with the Vo increment and disorder-to-order transition. To investigate the subject-guest interaction mechanism, three LSMO films (thickness, ~55 nm) initially grown on [001]-oriented SrTiO_3_ substrates under an oxygen partial pressure of *P*_O2_ = 1.0, 1.3, and 1.5 mbar were treated in NH_3_ plasma at 600°C for 2 hours. With a formula of N*_x_*H*_y_*La_0.7_Sr_0.3_MnO_3-δ_ (NH-LSMO), the samples are termed as IP1, IP2, and IP3 hereafter, respectively.

With respect to the as-grown (AG) sample grown under *P*_O2_ = 1.5 mbar, our x-ray diffraction (XRD) shows that after the NH_3_ plasma treatment, the IP1, IP2, and IP3 samples exhibit one-, four-, and twofold modulation along the pseudocubic *c*_p_ axis (Fig. 1, B and C). According to the structural modulation feature of LSMO (*11*, *12*), one knows that the three samples have a vacancy content of δ ≈ 0.125, 0.25, and 0.5, respectively. Referring to the *P*_O2_ growth condition, this reveals that the fresh LSMO sample with sufficient and deficient oxygen occupancy interacts more intense and inert with the NH_3_ plasma, which leads to more and less Vos, respectively. Along with the presence of the tetrahedral MnO_4_ units, where the Mn ionic radius becomes larger due to the valency reduction (Mn^3.3+^ → Mn^2+^), the octahedral distortion is enhanced, and the *c*_p_ axis is elongated by the increased Vo concentration (Fig. 1D). Accordingly, the lattice tetragonality is observed to increase from *c*_p_/*a*_p_ = 0.986 to 1.053 with the Vo content, and the enhanced lattice distortion creates a favorable condition for the emergence of crystallographic polarity in the NH-LSMO samples.

To examine the N-H injection behavior, depth-dependent secondary ion mass spectrometry (SIMS) experiments were performed. By calibrating the hydrogen content using mica, we find that the hydrogen unavoidably appears in the AG LSMO film, with *y* = 0.33. Apart from certain compositional fluctuation near the surface, we found that the hydrogen infiltrates quickly throughout the film, and its concentration scales almost linearly with the *P*_O2_ value, with *y* ≈ 0.54, 0.70, and 0.93 in the IP1, IP2, and IP3 samples, respectively (Fig. 1F). In contrast, accumulation of nitrogen at the upper half of each sample indicates a much sluggish incorporation process (Fig. 1G). As for the N/H ratio, one easily finds suppression of N injection by the H filling, which is contrary to our common sense on the demand of more protons (H^+^), while more O^2−^ ions are replaced by the N^3−^ ions.

Since the topotactic transformation easily yields a metal-insulator transition (*13*, *14*), we measured the film orientation–dependent resistivity change, which relates to the adjacency relation of octahedral- and tetrahedral-unit layers in the IP2 and IP3 samples. Along the in-plane direction, we found that the three samples have approximate resistivity, whose average value is 〈*R*_IP_〉 ≈ 17.5 kilohm. Along the out-of-plane direction, the resistivity of IP1 and IP3 samples increases by four times to *R*_OOP_ ≈ 74.7 kilohm. While for the IP2 sample, this value is enhanced by two orders of magnitude, with *R*_OOP_ = 3.0 megohm. This reveals a vacancy modulation–induced strong anisotropy of resistivity in the BM″ phase, which is quite weak in the PVL and BM phases possibly due to more disordered Vo (Fig. 1E).

### Identification of stabilized polar orders

**Fig. 2.**
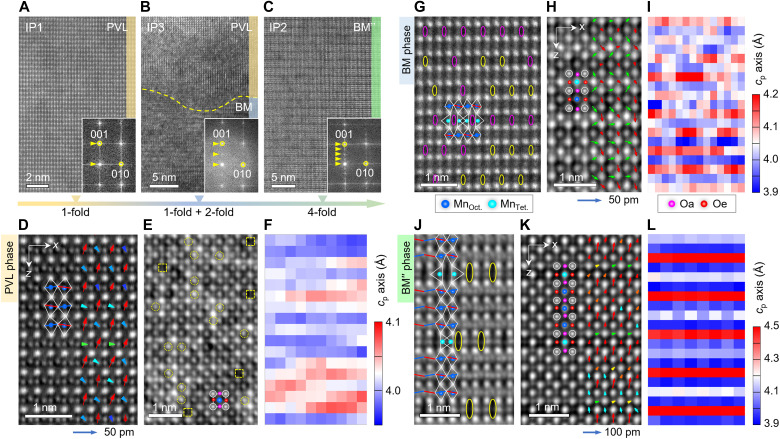
Octahedral distortion induced polarity. (**A** to **C**) Morphology of IP1, IP3, and IP2 samples and their FFT images (insets) along [100]_p_ direction of the PVL, BM, and BM” phases, respectively. (**D** to **F**) Reversed ABF and iDPC images of the PVL phase from the IP3 sample collected along [110]_p_ and [100]_p_ directions and the *c*_p_ axis map of (D), respectively. The dashed circles and squares in (E) mark the (A_Mn_, A/B_O_) antisite defects and random Vos. (**G** to **I**) High-angle annular dark field (HAADF) and iDPC images of the BM phase along [110]_p_ and [100]_p_ and the *c*_p_ axis map of (G), respectively. (**J** to **L**) The iDPC images of the BM” phase (collected Δ*t* = 0 min) along [110]_p_ and [100]_p_ and the *c*_p_ axis map of (K), respectively. The Oa (pink) and Oe (red) displacements are measured against A-site columns and overlaid on (D), (H), and (K), respectively. The elliptics and line segments in (G) and (J) denote the separated Mn-Mn columns and octahedral tilting, respectively.

To explore the N-H codoping induced structural changes, cross-sectional lamellae specimens are prepared for scanning transmission electron microscopy (STEM) observations (fig. S1). As the fast Fourier transform (FFT) images show (Fig. 2, A to C and their insets), the IP1 and IP2 samples exhibit nearly uniform PVL and BM” phases, respectively. While the IP3 sample consists of the PVL and BM phases with a phase ratio of ~1:1. This reveals that broadening of the SIMS nitrogen peak in the IP3 sample is attributed to the PVL phase. In other words, the disordered Vo distribution provides a favorable condition for the N-to-O substitution, which is supported by the shallowest penetration depth of nitrogen in the fourfold modulated BM” phase. Further, atom-resolved annular bright field (ABF) (*15*) and integrated differential phase contrast (iDPC) (*16*–*18*) STEM images are collected for probing the polarity-related structural motifs, e.g., the relative displacement of oxygen with respect to the heavy elements.

For the PVL phase in the IP1 and IP3 samples, our mapping-based positional analysis reveals that the equatorial oxygen (Oe) exhibits strongly asymmetric *z*-direction shifts, with δ*z*_Oe_ ≈ 24.8 pm and −2.8 pm, against centers of the nearest-neighboring A-site atoms (fig. S2), which is accompanied with an in-plane shift by δ*x*_Oe_ ≈ 4.3 pm. As manifested by the displacement vectors and profiles of 〈δ*z*_Oe_〉 and 〈δ*x*_Oe_〉, this demonstrates breaking of spatial inversion symmetry in the PVL phase (Fig. 2D and fig. S3A). Since the polarity arises from vacancy-induced octahedral distortion, the nonpolar-polar transition can be ascribed to the antiferrodistortive (AFD)– to–FD transition in terms of the phase transition theory (*19*–*22*). An iDPC image collected along [100]_p_ shows that apart from the polar shifts of oxygen, random Vos and antisite defects appear in the PVL phase as well. This leads to variation of the *c*_p_ axis in an irregular manner (Fig. 2, E and F).

As for the BM phase in the IP3 sample, our measurement on Mn-Mn separation reveals that instead of a long-range ordering, the tetragonal MnO_4_ pairs exhibits in-plane intertwining at the nanoscale (Fig. 2G). By analyzing oxygen displacements from the iDPC image, we find that the apical oxygen (Oa; green arrows) exhibits antipolar shifts along *z* (δ*z*_Oa_) direction, being analogous to the BM phase of SrCoO_2.5_ (*23*), and the Oe atom (red arrows) undergoes a net shift by 〈δ*z*_Oe_〉 = 23.5 ± 7.6 pm (Fig. 2H and fig. S3B). With partial preservation of the *c*_p_-axis modulation (Fig. 2I), we are aware that the polar order observed in the BM phase is distinct from the FE Pmc21 phase predicted by first-principles calculations, where only the in-plane polarization is permitted (*24*, *25*).

Referring to the BM phase, the atomically resolved iDPC image clearly verifies that the BM” phase is characteristic of a unique building block, i.e., one tetrahedral (1T) layer plus three octahedral (3O) layers, which shift by half a period along in-plane and one period along out-of-plane direction and form a unit cell (Fig. 2, J and K). By measuring the La/Sr positions from the iDPC images, we find that the *c*_p_ axis unexpectedly varies between 3.873 and 4.402 Å, up to 13.7% change. More astonishingly, two Oa-site atoms, linking the MnO_4_-MnO_6_ and the rest two MnO_6_ units, undergo a huge polar shift by 〈δ*z*_Oa_〉 = 56.8 ± 6.2 pm, and the Oe atoms shift by 〈δz_Oe_〉 = 18.9 ± 6.3 pm, where their in-plane shifts are within the measurement error (fig. S3C). Different from the ordinary FE insulators and FE metals (*26*, *27*), this indicates that the BM” phase has a q2D character, which is supported by the ultralarge interlayer spacing and the strongly anisotropic resistivity.

### q2D ferroelectricity and multiple polarization states

**Fig. 3.**
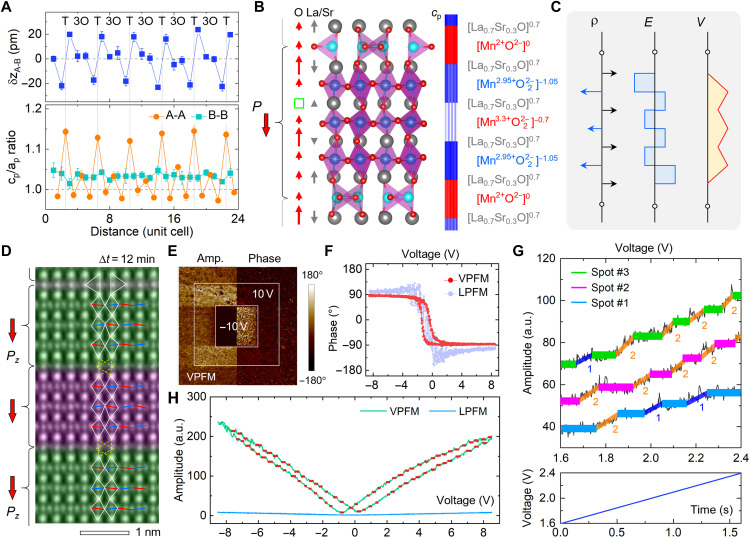
Evidence of q2D ferroelectricity and multiple polarization states in the BM” phase. (**A**) A-site atom shifts against centers of B-site atoms along *z* axis and *c*_p_/*a*_p_ ratio measured from A-A and B-B columns, respectively. (**B**) Displacements of oxygen (red arrow) and La/Sr (gray arrow) atoms along the *z* axis, squeezed *c*_p_ axis map of Fig. 2L, and valence state in the BM” phase. (**C**) Atomic layer–dependent charge (ρ), electric field (*E*), and electric potential (*V*) distribution in an 1T-3O q2D layer. (**D**) An iDPC image taken at Δ*t* = 12 min along [110]_p_ direction showing the electron beam–induced collective retilting of the 3O units (purple shaded region). (**E**) Vertical (V) and lateral (L) PFM amplitude and phase images poled and switched by ±10-V voltage with the total scanning area of 15 μm by 15 μm. (**F**) Corresponding V-PFM and L-PFM phase loops. (**G**) Step-wise V-FPM amplitude hopping curves (top) measured from different sample regions and time dependence of the applied voltage (bottom). The 1 and 2 denote the switching number of q2D layers. (**H**) Step-wise V-PFM amplitude hopping and ignorable L-PFM amplitude in the whole hysteresis loops.

To elaborate the q2D attribute, further structural motifs of the BM” phase are analyzed. With respect to the Mn atoms, we found that the A-A atoms bracing the MnO_4_ layers shift in antiparallel by δz_A-B_ ≈ ± 19.1 pm, which yields the exceptionally large 〈*c*_p_/*a*_p_〉_A_ = 1.131. In sharp contrast, a constant value is measured from the B-B atoms, with 〈*c*_p_/*a*_p_〉_B_ = 1.033 ± 0.008 (Fig. 3A). This indicates that the B-site cations serve as the rigid lattice skeleton for flexible movement of the A- and O-site atoms. Since the lattice parameter changes are governed by Vo-based interlayer electrostatic force, e.g., the repulsive [La_0.7_Sr_0.3_O]^0.7+^ planes linked by the electroneutral [MnO]^0^ plane (*28*–*30*), this shows that the Mn valence state in each pseudocubic unit cell can be derived from the interlayer spacing of A-A columns along *z* direction. By linearly scaling the relationship of *c*_p_-axis length with the net charge value per pseudocubic unit cell, one can obtain the valence states of Mn ions, which are Mn^2+^, Mn^2.95+^, Mn^3.3+^, and Mn^2.95+^ in the 1T-3O building block, respectively (Fig. 3B and fig. S4).

On this basis, one can plot an alternating interlayer electric field (*E*) and a sawtooth-like electric potential (*V*) for each 1T-3O building block, which is derived from the charge density (ρ) of each atomic plane (Fig. 3C). This accounts for the q2D attribute of the 1T-3O building blocks in the BM” phase, which has an ideal stoichiometry of La_0.7_Sr_0.3_MnO_2.75_. According to the coupled polarization-strain relationship in ordinary FEs (*31*), i.e., *P*^2^ = κ (*c*/*a* − 1), our evaluation suggests that the mean polarization of the BM” phase is 〈*Pz*〉 = 63.7 μC/cm^2^, where the maximum polarization is up to *Pz*_max_ = 124.2 μC/cm^2^ for the unit cell layer with the largest *c*/*a* ratio (fig. S5). In comparison, the polarization for the BM and Grenier phases of Nd_1/3_Ca_2/3_FeO_2.67_ and SrCoO_2.5_ (*24*, *25*, *32*, *33*) is in the range of 2 to 9 μC/cm^2^, which is one order of magnitude lower.

The q2D (1T-3O) layers in the BM” phase are validated by observation of collective retilting of the 3O layers as the specimen is irradiated by the scanning electron beam, where their initial out-of-phase octahedral tilting (Δ*t* = 0 min, angle θ_1,2_ ≈ 5.2° and 8.8°) is changed to an in-phase tilting (Δ*t* = 12 min, angle θ_1,2_ ≈ 2.6° and 4.1°). This is highlighted by red and blue line segments overlaid on the images (Figs. 2J and 3D and fig. S6). By analyzing the Oe shifts along the *z* axis, we find that the octahedral retilting decouples with the polarization switching, which is possibly assisted by oxygen filling at the vacant sites in the tetrahedral layers, e.g., transforming into a PVL phase by forming a continuous polarization configuration. Otherwise, the electrically neutral [MnO]^0^ planes will act as charged interfaces about polarization, which are disfavored because of their extremely high formation energy (*34*). Since the 3O layers are less resistive than the T layers, as evidenced in H*_x_*SrCoO_2.5_, LSMO, and SrVO_2.2_N_0.6_ (*9*, *35*, *36*), the self-compensated out-of-plane polarization is naturally stabilized in the BM” phase. This makes it analogous to the 2D FE metal WTe_2_, which is electrically conductive at the 2D plane while the spontaneous polarization is normal to this plane (*37*).

As examining the switchability of polarization in the NH-LSMO samples, our piezoresponse force microscopy (PFM) experiments disclose more interesting findings. Being consistent with our STEM results, the polarization in IP1, IP2, and IP3 samples can be poled and switched by the applied electric field (Fig. 3E and fig. S7). However, different from the Vo-based resistive attributes of IP1 and IP3 samples, which are manifested by linear PFM responses and quick polarization decay, FE-like amplitude and phase hysteresis loops are observed in the IP2 sample. Furthermore, robust polarization retention is observed in our time-dependent PFM experiments (Fig. 3, F and H, and fig. S8), which demonstrates that the BM” phase is FE. Spectacularly, we find that the vertical (V) PFM (VPFM) amplitude loop exhibits a series of step-type jumping changes, denoting switchable multiple polarization states along *c* axis and ignorable lateral (L) PFM signals. Such a piezoresponse behavior is distinct from that of ordinary and inverse size-scaling FEs, where the field-dependent amplitude variation is continuous rather than discrete (*26*, *38*).

To further confirm the q2D feature of the 1T-3O building blocks, the VPFM amplitude signal is detected in a narrower window with finer voltage step size. In the 1.6- to 2.4-V measurement interval, which takes a time of 1.6 s, step-type jumping of amplitude response is reliably reproduced in different sample regions. At the same time, a narrow amplitude peak is frequently seen before reaching the stable plateau, and a short phase step can be vaguely identified as the amplitude jumps between different steps (Fig. 3G and fig. S9). Our statistical analysis reveals that the jumping amplitude varies in the range of 4.0 to 7.4 pm (SD = 1.4 pm), the mean voltage duration is *V*_d_ = 0.12 ± 0.03 V, and the jumping voltage covers *V*_j_ = 0.07 ± 0.02 V. By counting the total number of q2D layers in the IP2 sample, our evaluation yields that the amplitude change is in the range of 3.7 to 5.0 pm per single layer. This suggests that the step-wise polarization switching takes place in either one-layer or two-layer mode during our PFM experiments. One can naturally envisage that the q2D-layer–dependent polarization switching may correlate intimately with the following factors, e.g., retilting of 1T-3O units, interlayer lattice expansion and contraction, structural defects, dynamic migration of Vos and protons, etc.

### Direct N-H imaging at atomic resolution

**Fig. 4.**
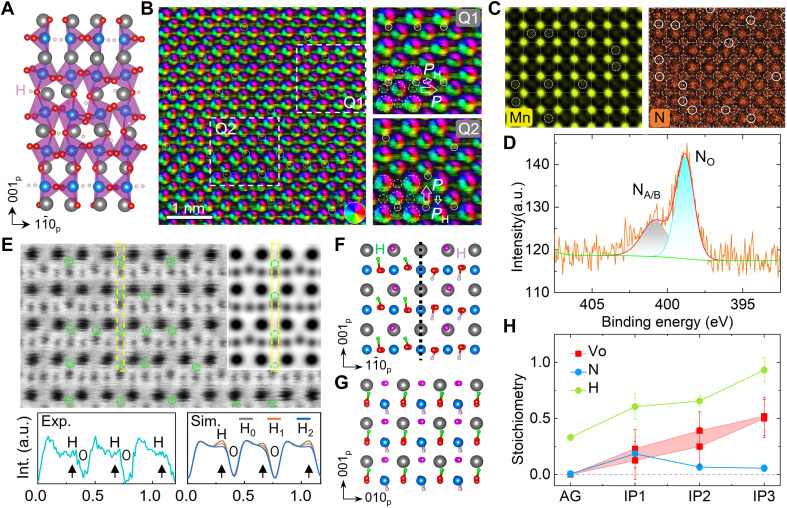
Occupancy of H and N in the atomic lattices. (**A**) H-intercalated BM” phase obtained by first-principles calculations. (**B**) DPC image of the PVL phase collected along [100]_p_ direction and magnified Q1 and Q2 regions with marking of the H locations (solid circles). The H^+^-induced electric dipoles are denoted by *P*_H_ with respect to the H-free unit cell dipole *P*. (**C**) Atomic-resolution EDS maps of Mn and N with annotation of Mn_La/Sr_ antisites (dashed circles) and occupancy of N at La/Sr, Mn, and O sites in the PVL phase, respectively. (**D**) X-ray photoelectron spectroscopy N-1s spectrum collected from the IP2 sample. (**E**) Experimental (Exp.), simulated (Sim.). ABF (inset, thickness = 15 nm, defocus = 0 nm) images of the PVL phase along [110]_p_ direction and comparison of the intensity (Int.) profiles extracted from the atomic rows (yellow dashed bar). (**F** and **G**) Structural models of H-LSMO with H^+^ coordinating with different Oe atoms (red) viewed along [110]_p_ (top) and [100]_p_ direction (bottom), respectively. (**H**) Plot of Vo, N, and H stoichiometry deduced from the SIMS, EDS, and XRD data of the NH-LSMO samples.

To better understand the above results, we probe the location of N and H in the NH-LSMO samples. First-principles calculations on the BM” phase of H_0.75_La_0.7_Sr_0.3_MnO_2.75_ show that the hydrogen tends to form charge neutral H-H dimers at the MnO_4_ layers and Oa-H^+^ bonds (Fig. 4A), which is consistent with the result reported on H*_x_*SrCoO_2.5_ (*35*). By using differential phase contrast (DPC) microscopy technique (*39*) to the PVL phase, we find that the H atoms can be directly resolved by detecting the electric field distribution. Along the projected [100]_p_ plane, the H atoms are found to locate at the bridging interstitial sites of La/Sr-Oe bonds (Q1 region) and Mn-Oa bonds (Q2 region), respectively (Fig. 4B). Accordingly, the lattice tetragonality is measured as *c*_p_/*a*_p_ ≈ 1.014 and 1.067, and the H-induced morphotropic polar domains or phases are manifested by the large oxygen displacements along in-plane and out-of-plane directions, respectively. By comparing with the H-free models, one can easily find out that the intercalated H enhances and inhibits the polarization along these two directions, respectively (fig. S10).

For the PVL phase with an [110]_p_ orientation, the hydrogen location is probed by using the ABF-STEM technique (Fig. 4E). Featured by weak dark contrast (green circles), the hydrogen atoms are resolved at the bridging interstitials of A-Oe bonds along the diagonal direction, which is supported by our image simulations. By comparing the intensity profiles extracted from the marked regions, it is suggested that the H content is around one atom per pseudocubic unit cell. As a whole, drawing of the 3D structure shows that the H essentially coordinates with the Oe atoms in the form of proton, as the models illustrated (Fig. 4, F and G). Since the Oe-H bonds point upward and downward at the Q1 and Q2 regions, respectively, it is speculated that orientational domains can be generated by H atoms locating at different interstitial sites.

Benefitting from the superior stability of the electron microscopy, apart from H, all the elements are successfully resolved by our energy dispersive x-ray spectroscopy (EDS). Besides fractional antisite defects in the NH-LSMO samples, e.g., La/Sr_Mn_ and Mn_La/Sr_ (dashed circles), we observe that the N locates not only at the O site (solid circles) but also majorly at the La/Sr and Mn sites (dashed circles), which is substantiated by the EDS data of the N-free LSMO sample (Fig. 4C and fig. S11). This tells that fractional cations are lost during the plasma treatment. Furthermore, the structural disordering, as manifested by N_La/Sr_ and N_Mn_ antisite defects, is enhanced, which should play a positive role in stabilizing the polar orders observed in different structural phases. In combination with the SIMS data, our analysis yields that the N content is *x* ≈ 0.19, 0.07, and 0.06 in the IP1, IP2, and IP3 samples, respectively (Fig. 4H). By probing the binding energy of N using x-ray photoelectron spectroscopy, we find that two N 1s peaks appearing at 398.7 and 400.8 eV can be assigned to N occupancy at the O site and A/B site, respectively (Fig. 4D). The binding energy-valency relationship suggests that the N valency is in the range of −3.0 to −1.5 (fig. S12).

## DISCUSSION

Previous in situ electron microscopy studies have reported that the electric field can trigger the PV-BM and metal-insulator transitions in LSMO, which is dominated by the reversible Vo migration (*14*, *40*). In comparison, our results show that the N-H coinjection adds a new degree of freedom, i.e., electric polarization, during the phase transition. As for their BM precursor phase (*c*_p_ ≈ 0.415 nm) appearing at ≥3.0 V, our data suggest that this is probably the FD PVL phase (*c*_p_ ≈ 0.412 nm) that may tolerate a different Vo content (δ ≈ 0.125 to 0.5). Apart from the degree of Vo ordering that governs the PV-BM transitions, the antisite-type structural defects should be considered since they play an important role in trigging the nonpolar-polar transition (*26*), as observed in all the NH-LSMO samples. With respect to the successful synthesis of LaWN_3_ (*8*), one can naturally foresee that the cationic valency predominantly determines the N-to-O substitution amount. Besides, although the H facilitates the N diffusion, our results unveil that the entire process is greatly suppressed by the Vos. This implies that the N substitution at the cationic sites is a compromise between multiple competitions, e.g., chemical, electronic, polarization, and lattice degrees of freedom.

When it comes to the most interesting BM” phase, on the one hand, the structure and valency model suggests that the fractional N occupancy at cationic sites may lower the electrostatic energy of the whole system (Fig. 3B). Specifically, the slightly negatively charged [N-MnO]^δ−^ planes may accommodate more intercalated H^+^. On the other hand, the enhanced proton conductivity, as documented in H*_x_*SrCoO_2.5_ (*4*), may possibly help to better stabilize the large out-of-plane polarization in the q2D layers, whose origin can be ascribed to the atomic antisite defects as well. Compared with the H doping–induced dipolar polarization in H*_x_*NdNiO_3_, which quickly decays within ~1 s (*41*), our systematic results point out that controlling the Vo-regulated structural modulation period plays an important role in stabilizing the long-range electric polarization in the N-H coinjected perovskite oxides.

In summary, our systematic study on NH-LSMO thin films points out that the topotactic treatment via NH_3_ plasma induces unexpected structural phases with intriguing AFD-FD phase transition, which is driven by the presence of atomic antisite defects. Governed by different degrees of Vo ordering, our study indicates that the N and H coinjection can help to stabilize the high-fold structural modulation and out-of-plane electric polarization, respectively. Spectacularly, a q2D FE phase, consisting of 1T-3O building blocks with a fourfold structural modulation, is unveiled to have switchable series of polarization states by an applied electric field. Distinct from the available strategies for storing information, e.g., magnetic skyrmion-based artificial synapses (*42*), Majorana zero modes (*43*), quantum memory with cold atoms (*44*), and even sliding FEs (*45*), our findings present that the N-H coinjected manganites provide a unique platform for such a potential device application, once the Vo ordering can be engineered in a controllable manner.

## MATERIALS AND METHODS

### Material growth

Epitaxial La_0.7_Sr_0.3_MnO_3-δ_ thin films were grown on atomically flat (001) SrTiO_3_ substrates by high-order photonic structure deposition using a sputter target with the corresponding chemical stoichiometry. The AG samples were deposited at a substrate temperature of 800°C and oxygen partial pressures of *P*_O2_ = 1.0, 1.3, and 1.5 mbar, respectively. Following this, the fresh thin-film samples were annealed at 600°C for 2 hours under the NH_3_ plasma atmosphere. The crystalline structure of the LSMO films was characterized by a Bruker D8 x-ray diffractometer using monochromatic Cu *K*_α1_ radiation (λ = 1.5405 Å).

### Scanning transmission electron microscopy

Cross-sectional lamella specimens were cut by using a focused ion beam (FEI Helios NanoLab 400S) system, which were then milled down by using Ga ion operated at a voltage reducing from 30 to 5 kV and the ionic beam current reducing from 2.8 nA to 90 pA. NanoMill (model 1040) operated at 500 V was used to mill down and remove the surface contamination. A FEI Titan 80-200 ChemiSTEM microscope was used to collect the HAADF, ABF, and iDPC STEM images with a semiconvergent angle of 24.7 mrad. A JEOL ARM 300F2 microscope, equipped with a couple of ultralarge area (316 mm^2^) silicon-drift detector (SDD) system, was used to collect the DPC images in an angular range of 12 to 24 mrad and the EDS data. To enhance the signal-to-noise ratio, certain subframe ABF and iDPC images are stacked to increase the positional measurement accuracy. The atom column positions were measured by fitting column peak intensity using 2D Gaussian function–based maximum likelihood estimation. Dr. Probe was used for image simulations, and VESTA software package was used for drawing the crystal structure.

### Piezoresponse force microscopy

The PFM measurements were carried out on an atomic force microscope (Bruker Nano Inc., Dimension FastScan, USA) at the single frequency resonance tracking mode. Conductive Co/Cr-coated silicon cantilevers (model MESP-RC-V2, with the resonance frequency of 150 kHz and a force constant of ~5 N/m) were used for both PFM imaging and hysteresis loop measurement. In our PFM measurement, the thin-film samples were transferred onto the conductive sample stage. A conductive tip was driven with an ac voltage of *U*_ac_ = 0.5 to 1 V under the tip-sample contact resonant frequency. The resonance frequency of the FE test was measured as *f* ≈ 600 kHz. The PFM measurements were carried out under a constant temperature and dry condition.

### Time-of-flight secondary ion mass spectroscopy

The time-of-flight–SIMS data were obtained using an IONTOF M6 instrument, in which the mass resolution is about 200 atomic mass units. The Cs ion sputtering beam (1 keV) was used to produce a crater with a size about 500 μm by 500 μm, and the negative secondary ions were collected from a central analysis area (100 μm by 100 μm) to minimize disturbances emanating from the edge of the crater. The natural mica, with a known bulk density of H atoms (~0.853 × 10^22^ atom cm^−3^), was used as a reference sample to quantify the hydrogen content. The bulk density of H atoms in the LSMO series samples was derived by comparing the SIMS results with the natural mica, where their unit cell parameters are used for calculating the H content on the single-unit cell scale.

### Resistivity measurement

The silver glue was pasted on surfaces and sides of the IP1, IP2, and IP3 materials to measure the voltammetry curves of the materials. The probe station for semiconductor device measurement (Lake Shore CRX-6.5K) and the supporting probe were used to measure the voltammetry curves of the materials. By controlling Keithley Instruments (model 4200) semiconductor characterization system, we apply cyclic voltages of −0.5 to 0.5 V, −1 to 1 V, −3 to 3 V, −5 to 5 V, and −10 to 10 V to the material, respectively. The corresponding *I*-*V* curve is obtained for calculating the resistivity.

## References

[1] Z. Meng, H. Yan, P. Qin, X. Zhou, X. Wang, H. Chen, L. Liu, Z. Liu, Topotactic transition: A promising opportunity for creating new oxides. Adv. Funct. Mater. 33, 2305225 (2023).

[2] N. Lu, P. Zhang, Q. Zhang, R. Qiao, Q. He, H.-B. Li, Y. Wang, J. Guo, D. Zhang, Z. Duan, Z. Li, M. Wang, S. Yang, M. Yan, E. Arenholz, S. Zhou, W. Yang, L. Gu, C.-W. Nan, J. Wu, Y. Tokura, P. Yu, Electric-field control of tri-state phase transformation with a selective dual-ion switch. Nature 546, 124–128 (2017). 28569818 10.1038/nature22389

[3] L. Li, M. Wang, Y. Zhou, Y. Zhang, F. Zhang, Y. Wu, Y. Wang, Y. Lyu, N. Lu, G. Wang, H. Peng, S. Shen, Y. Du, Z. Zhu, C. W. Nan, P. Yu, Manipulating the insulator-metal transition through tip-induced hydrogenation. Nat. Mater. 21, 1246–1251 (2022). 36175522 10.1038/s41563-022-01373-4

[4] N. Lu, Z. Zhang, Y. Wang, H.-B. Li, S. Qiao, B. Zhao, Q. He, S. Lu, C. Li, Y. Wu, M. Zhu, X. Lyu, X. Chen, Z. Li, M. Wang, J. Zhang, S. C. Tsang, J. Guo, S. Yang, J. Zhang, K. Deng, D. Zhang, J. Ma, J. Ren, Y. Wu, J. Zhu, S. Zhou, Y. Tokura, C.-W. Nan, J. Wu, P. Yu, Enhanced low-temperature proton conductivity in hydrogen-intercalated brownmillerite oxide. Nat. Energy 7, 1208–1216 (2022).

[5] X. Ding, C. C. Tam, X. Sui, Y. Zhao, M. Xu, J. Choi, H. Leng, J. Zhang, M. Wu, H. Xiao, X. Zu, M. Garcia-Fernandez, S. Agrestini, X. Wu, Q. Wang, P. Gao, S. Li, B. Huang, K. J. Zhou, L. Qiao, Critical role of hydrogen for superconductivity in nickelates. Nature 615, 50–55 (2023). 36859583 10.1038/s41586-022-05657-2

[6] S. T. Hartman, A. S. Thind, R. Mishra, Tin oxynitride-based ferroelectric semiconductors for solar energy conversion applications. Chem. Mater. 32, 9542–9550 (2020).

[7] C. Lawley, M. Nachtegaal, J. Stahn, V. Roddatis, M. Dobeli, T. J. Schmidt, D. Pergolesi, T. Lippert, Examining the surface evolution of LaTiO_x_N_y_ an oxynitride solar water splitting photocatalyst. Nat. Commun. 11, 1728 (2020). 32265498 10.1038/s41467-020-15519-y PMC7138824

[8] K. R. Talley, C. L. Perkins, D. R. Diercks, G. L. Brennecka, A. Zakutayev, Synthesis of LaWN_3_ nitride perovskite with polar symmetry. Science 374, 1488–1491 (2021). 34914511 10.1126/science.abm3466

[9] T. Yamamoto, A. Chikamatsu, S. Kitagawa, N. Izumo, S. Yamashita, H. Takatsu, M. Ochi, T. Maruyama, M. Namba, W. Sun, T. Nakashima, F. Takeiri, K. Fujii, M. Yashima, Y. Sugisawa, M. Sano, Y. Hirose, D. Sekiba, C. M. Brown, T. Honda, K. Ikeda, T. Otomo, K. Kuroki, K. Ishida, T. Mori, K. Kimoto, T. Hasegawa, H. Kageyama, Strain-induced creation and switching of anion vacancy layers in perovskite oxynitrides. Nat. Commun. 11, 5923 (2020). 33230157 10.1038/s41467-020-19217-7 PMC7683707

[10] Y. Xing, I. Kim, K. T. Kang, J. Byun, W. S. Choi, J. Lee, S. H. Oh, Monitoring the formation of infinite-layer transition metal oxides through in situ atomic-resolution electron microscopy. Nat. Chem. 17, 66–73 (2024). 39191854 10.1038/s41557-024-01617-7 PMC11976294

[11] L. Cao, O. Petracic, P. Zakalek, A. Weber, U. Rücker, J. Schubert, A. Koutsioubas, S. Mattauch, T. Brückel, Reversible control of physical properties via an oxygen-vacancy-driven topotactic transition in epitaxial La_0.7_Sr_0.3_MnO_3-δ_ thin films. Adv. Mater. 31, 1806183 (2019). 10.1002/adma.201806183 30570780

[12] T. G. Parsons, H. D’Hondt, J. Hadermann, M. A. Hayward, Synthesis and structural characterization of La1−xAxMnO2.5 (A = Ba, Sr, Ca) phases: Mapping the variants of the brownmillerite structure. Chem. Mater. 21, 5527–5538 (2009).

[13] L. Cao, O. Petracic, X. K. Wei, H. Zhang, T. Duchoň, F. Gunkel, A. Koutsioubas, K. Zhernenkov, K. Z. Rushchanskii, H. Hartmann, M. Wilhelm, Z. Li, Y. Xie, S. He, M. L. Weber, K. Veltruská, A. Stellhorn, J. Mayer, S. Zhou, T. Brückel, Migration kinetics of surface ions in oxygen-deficient perovskite during topotactic transitions. Small 17, 2104356 (2021). 10.1002/smll.202104356 34791798

[14] L. Yao, S. Inkinen, S. van Dijken, Direct observation of oxygen vacancy-driven structural and resistive phase transitions in La_2/3_Sr_1/3_MnO_3_. Nat. Commun. 8, 14544 (2017). 28230081 10.1038/ncomms14544 PMC5331213

[15] S. D. Findlay, N. Shibata, H. Sawada, E. Okunishi, Y. Kondo, Y. Ikuhara, Dynamics of annular bright field imaging in scanning transmission electron microscopy. Ultramicroscopy 110, 903–923 (2010). 20434265 10.1016/j.ultramic.2010.04.004

[16] I. Lazic, E. G. T. Bosch, S. Lazar, Phase contrast STEM for thin samples: Integrated differential phase contrast. Ultramicroscopy 160, 265–280 (2016). 26590505 10.1016/j.ultramic.2015.10.011

[17] I. Lazic, M. Wirix, M. L. Leidl, F. de Haas, D. Mann, M. Beckers, E. V. Pechnikova, K. Muller-Caspary, R. Egoavil, E. G. T. Bosch, C. Sachse, Single-particle cryo-EM structures from iDPC-STEM at near-atomic resolution. Nat. Methods 19, 1126–1136 (2022). 36064775 10.1038/s41592-022-01586-0 PMC9467914

[18] B. Shen, H. Wang, H. Xiong, X. Chen, E. G. T. Bosch, I. Lazic, W. Qian, F. Wei, Atomic imaging of zeolite-confined single molecules by electron microscopy. Nature 607, 703–707 (2022). 35831496 10.1038/s41586-022-04876-x

[19] M. E. Lines, A. M. Glass, *Principles and Applications of Ferroelectrics and Related Materials*. (Oxford Univ. Press, 1977).

[20] X.-K. Wei, C. L. Jia, H. C. Du, K. Roleder, J. Mayer, R. E. Dunin-Borkowski, An unconventional transient phase with cycloidal order of polarization in energy-storage antiferroelectric PbZrO_3_. Adv. Mater. 32, 1907208 (2020). 10.1002/adma.201907208 31975474

[21] X.-K. Wei, R. E. Dunin-Borkowski, J. Mayer, Structural phase transition and in-situ energy storage pathway in nonpolar materials: A review. Materials 14, 7854 (2021). 34947446 10.3390/ma14247854 PMC8707040

[22] X.-K. Wei, C. L. Jia, K. Roleder, R. E. Dunin-Borkowski, J. Mayer, In situ observation of point-defect-induced unit-cell-wise energy storage pathway in antiferroelectric PbZrO_3_. Adv. Funct. Mater. 31, 2008609 (2021).

[23] Q. Zhang, X. He, J. Shi, N. Lu, H. Li, Q. Yu, Z. Zhang, L. Q. Chen, B. Morris, Q. Xu, P. Yu, L. Gu, K. Jin, C. W. Nan, Atomic-resolution imaging of electrically induced oxygen vacancy migration and phase transformation in SrCoO_2.5-δ_. Nat. Commun. 8, 104 (2017). 28740076 10.1038/s41467-017-00121-6 PMC5524633

[24] H. Tian, X.-Y. Kuang, A.-J. Mao, Y. Yang, H. Xiang, C. Xu, S. O. Sayedaghaee, J. Íñiguez, L. Bellaiche, Novel type of ferroelectricity in brownmillerite structures: A first-principles study. Phys. Rev. Mater. 2, 084402 (2018).

[25] Y. Shin, G. Galli, Tunable ferroelectricity in oxygen-deficient perovskites with Grenier structure. Npj Comput. Mater. 9, 217 (2023).

[26] X.-K. Wei, N. Domingo, Y. Sun, N. Balke, R. E. Dunin-Borkowski, J. Mayer, Progress on emerging ferroelectric materials for energy harvesting, storage and conversion. Adv. Energy Mater. 12, 2201199 (2022).

[27] X.-K. Wei, G. Bihlmayer, X. Zhou, W. Feng, Y. V. Kolen’ko, D. Xiong, L. Liu, S. Blugel, R. E. Dunin-Borkowski, Discovery of real-space topological ferroelectricity in metallic transition metal phosphides. Adv. Mater. 32, 2003479 (2020). 10.1002/adma.202003479 33029890

[28] Y. Hong, P. Byeon, J. Bak, Y. Heo, H. S. Kim, H. B. Bae, S. Y. Chung, Local-electrostatics-induced oxygen octahedral distortion in perovskite oxides and insight into the structure of Ruddlesden-Popper phases. Nat. Commun. 12, 5527 (2021). 34545102 10.1038/s41467-021-25889-6 PMC8452630

[29] S. Chen, H. Zhou, X. Ye, Z. Chen, J. Zhao, S. Das, C. Klewe, L. Zhang, E. Lupi, P. Shafer, E. Arenholz, D. Jin, H. Huang, Y. Lu, X. Li, M. Wu, S. Ke, H. Xu, X. Zeng, C. Huang, L. W. Martin, L. Chen, Versatile and highly efficient controls of reversible topotactic metal–insulator transitions through proton intercalation. Adv. Funct. Mater. 29, 1907072 (2019).

[30] J. Lee, Y. Ha, S. Lee, Hydrogen control of double exchange interaction in La_0.67_ Sr_0.33_MnO_3_ for ionic-electric-magnetic coupled applications. Adv. Mater. 33, e2007606 (2021). 33576067 10.1002/adma.202007606

[31] T. Qi, I. Grinberg, A. M. Rappe, Correlations between tetragonality, polarization, and ionic displacement in PbTiO_3_-derived ferroelectric perovskite solid solutions. Phys. Rev. B 82, 134113 (2010).

[32] K. T. Kang, C. J. Roh, J. Lim, T. Min, J. H. Lee, K. Lee, T. Y. Lee, S. Kang, D. Seol, J. Kim, H. Ohta, A. Khare, S. Park, Y. Kim, S. C. Chae, Y. S. Oh, J. Lee, J. Yu, J. S. Lee, W. S. Choi, A room-temperature ferroelectric ferromagnet in a 1D tetrahedral chain network. Adv. Mater. 31, e1808104 (2019). 31034128 10.1002/adma.201808104

[33] H. Li, Y. Yang, S. Deng, L. Zhang, S. Cheng, E.-J. Guo, T. Zhu, H. Wang, J. Wang, M. Wu, P. Gao, H. Xiang, X. Xing, C. Jun, Role of oxygen vacancies in colossal polarization in SmFeO_3-δ_ thin films. Sci. Adv. 8, eabm8550 (2022). 35363530 10.1126/sciadv.abm8550 PMC10938629

[34] P. S. Bednyakov, B. I. Sturman, T. Sluka, A. K. Tagantsev, P. V. Yudin, Physics and applications of charged domain walls. NPJ Computat. Mater. 4, 65 (2018).

[35] H. B. Li, F. Lou, Y. Wang, Y. Zhang, Q. Zhang, D. Wu, Z. Li, M. Wang, T. Huang, Y. Lyu, J. Guo, T. Chen, Y. Wu, E. Arenholz, N. Lu, N. Wang, Q. He, L. Gu, J. Zhu, C. W. Nan, X. Zhong, H. Xiang, P. Yu, Electric field-controlled multistep proton evolution in H_x_SrCoO_2.5_ with formation of H-H dimer. Adv. Sci. 6, 1901432 (2019). 10.1002/advs.201901432 PMC6794722 31637170

[36] E. J. Moon, P. V. Balachandran, B. J. Kirby, D. J. Keavney, R. J. Sichel-Tissot, C. M. Schleputz, E. Karapetrova, X. M. Cheng, J. M. Rondinelli, S. J. May, Effect of interfacial octahedral behavior in ultrathin manganite films. Nano Lett. 14, 2509–2514 (2014). 24697503 10.1021/nl500235f

[37] Z. Fei, W. Zhao, T. A. Palomaki, B. Sun, M. K. Miller, Z. Zhao, J. Yan, X. Xu, D. H. Cobden, Ferroelectric switching of a two-dimensional metal. Nature 560, 336–339 (2018). 30038286 10.1038/s41586-018-0336-3

[38] L. Chen, X. Ma, Z. Liang, Y. Wang, F. Liu, Y. Ma, Y. H. Bao, K. Q. Lin, Q. Li, B. Xu, X.-K. Wei, Inverse size-scaling ferroelectricity in centrosymmetric insulating perovskite oxide DyScO_3_. Adv. Mater. 37, 2413708 (2024). 10.1002/adma.202413708 39641180

[39] N. Shibata, S. D. Findlay, Y. Kohno, H. Sawada, Y. Kondo, Y. Ikuhara, Differential phase-contrast microscopy at atomic resolution. Nat. Phys. 8, 611–615 (2012).

[40] P. Nukala, M. Ahmadi, Y. Wei, S. d. Graaf, E. Stylianidis, T. Chakrabortty, S. Matzen, H. W. Zandbergen, A. Björling, D. Mannix, D. Carbone, B. Kooi, B. Noheda, Reversible oxygen migration and phase transitions in hafnia-based ferroelectric devices. Science 372, 630–635 (2021). 33858991 10.1126/science.abf3789

[41] Y. Yuan, M. Kotiuga, T. J. Park, R. K. Patel, Y. Ni, A. Saha, H. Zhou, J. T. Sadowski, A. Al-Mahboob, H. Yu, K. Du, M. Zhu, S. Deng, R. S. Bisht, X. Lyu, C. M. Wu, P. D. Ye, A. Sengupta, S. W. Cheong, X. Xu, K. M. Rabe, S. Ramanathan, Hydrogen-induced tunable remanent polarization in a perovskite nickelate. Nat. Commun. 15, 4717 (2024). 38830914 10.1038/s41467-024-49213-0 PMC11148064

[42] K. M. Song, J.-S. Jeong, B. Pan, X. Zhang, J. Xia, S. Cha, T.-E. Park, K. Kim, S. Finizio, J. Raabe, J. Chang, Y. Zhou, W. Zhao, W. Kang, H. Ju, S. Woo, Skyrmion-based artificial synapses for neuromorphic computing. Nat. Electron. 3, 148–155 (2020).

[43] C. Nayak, S. H. Simon, A. Stern, M. Freedman, S. Das Sarma, Non-Abelian anyons and topological quantum computation. Rev. Mod. Phys. 80, 1083–1159 (2008).

[44] X.-H. Bao, A. Reingruber, P. Dietrich, J. Rui, A. Dück, T. Strassel, L. Li, N.-L. Liu, B. Zhao, J.-W. Pan, Efficient and long-lived quantum memory with cold atoms inside a ring cavity. Nat. Phys. 8, 517–521 (2012).

[45] R. Bian, R. He, E. Pan, Z. Li, G. Cao, P. Meng, J. Chen, Q. Liu, Z. Zhong, W. Li, F. Liu, Developing fatigue-resistant ferroelectrics using interlayer sliding switching. Science 385, 57–62 (2024). 38843352 10.1126/science.ado1744

