# Supplementary material for: Quasi–two-dimensional ferroelectricity with multiple switchable polarization states in N-H coinjected perovskite manganites
Source: Sci Adv. 2025 Oct 1;11(40):eadx3747. doi: 10.1126/sciadv.adx3747 (PMC12487897; doi:10.1126/sciadv.adx3747)
Supplement: Supplementary file 1 — Figs. S1 to S12 [file sciadv.adx3747_sm.pdf]

Science Advances
AAAS

Supplementary Materials for

Quasi–two-dimensional ferroelectricity with multiple switchable polarization states in N-H coinjected perovskite manganites

Xian-Kui Wei *et al.*

Corresponding author: Xian-Kui Wei, xkwei@xmu.edu.cn; Lei Cao, caoleiinhit@gmail.com

Sci. Adv. **11**, eadx3747 (2025)
DOI: 10.1126/sciadv.adx3747

This PDF file includes:

Figs. S1 to S12

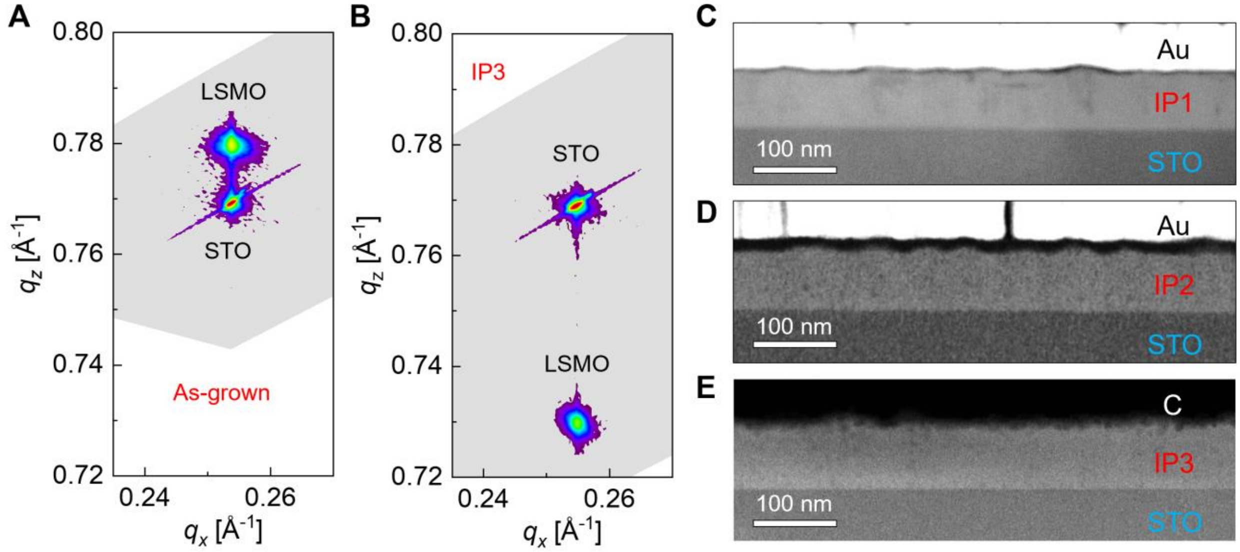

Fig. S1. Structural characterization of NH-LSMO samples. (**A,B**) Reciprocal space mapping of the as-grown and IP3 LSMO samples grown on STO substrates, respectively. (**C-E**) Cross-sectional HAADF STEM image of NH_3 plasma-treated IP1, IP2 and IP3 sample, respectively.

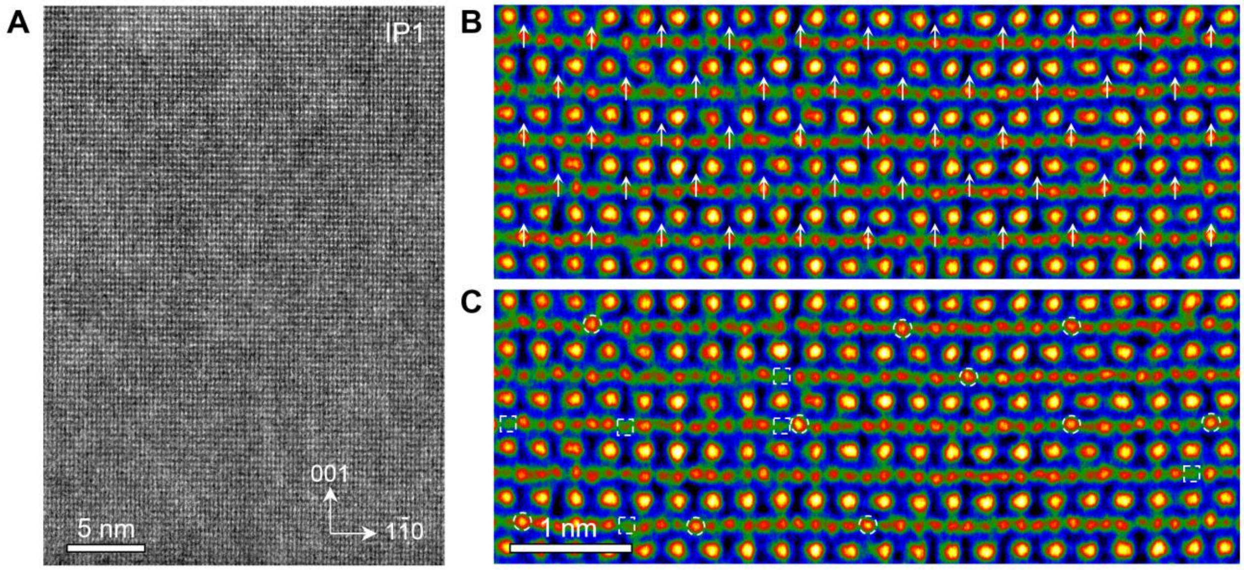

Fig. S2. FD structure in the IP1 sample. (**A**) HAADF-STEM image of IP1 sample viewed along $[110]_p$ direction. (**B**) False-color iDPC image with highlight of oxygen displacements with large amplitude along *z* direction. (**C**) The same iDPC image with highlight of probable vacancies at O and Mn sites (squares) and anti-occupancy of cations at the O site (circles), given the potential contrast reversal, tunable by the imaging parameter of defocus, in the phase-contrast iDPC image.

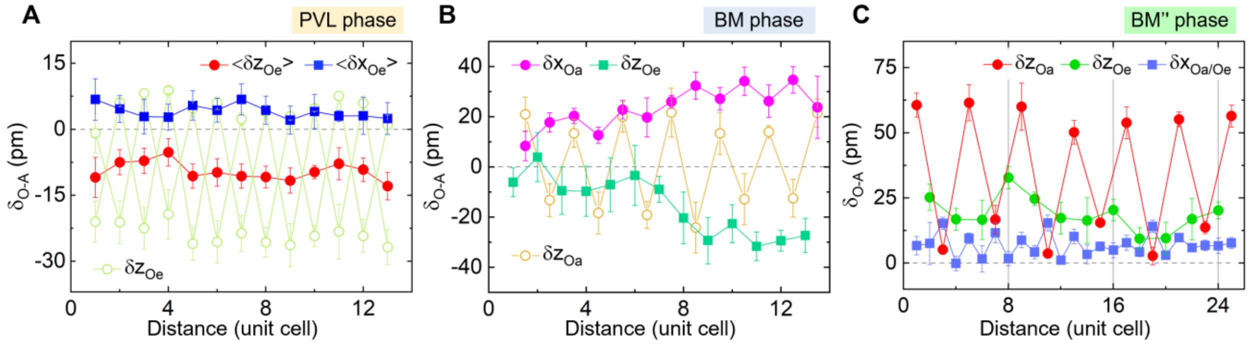

Fig. S3. Relative oxygen displacements against centers of the A-site cations. (**A**-**C**) Apical (Oa) and equatorial (Oe) oxygen shift profiles measured from (**D**) for the PVL phase, (**H**) for the BM phase, and (**K**) for the BM'' phase shown in Fig. 2.

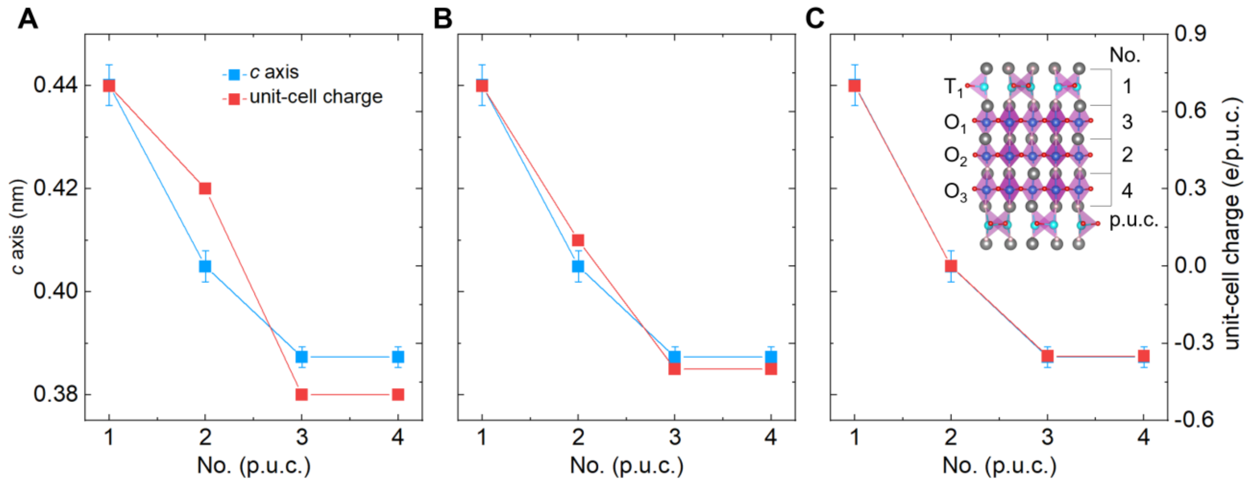

Fig. S4. Linear scaling of unit-cell charge with *c* axis in the BM'' phase. (**A-C**) The linear scaling relationship between c axis and charges per pseudocubic unit cell (p.u.c.) with consideration of Mn valence states at tetrahedral and octahedral sites from Mn_{T1}, Mn_{O1}, Mn_{O2} to Mn_{O3} as (**A**) 2+, 2.8+, 3.6+ to 2.8+, (**B**) 2+, 2.9+, 3.4+ to 2.9+, (**C**) 2+, 2.95+, 3.3+ to 2.95+. The inset in (**C**) illustrate the pseudocubic unit cell and the polyhedral units.

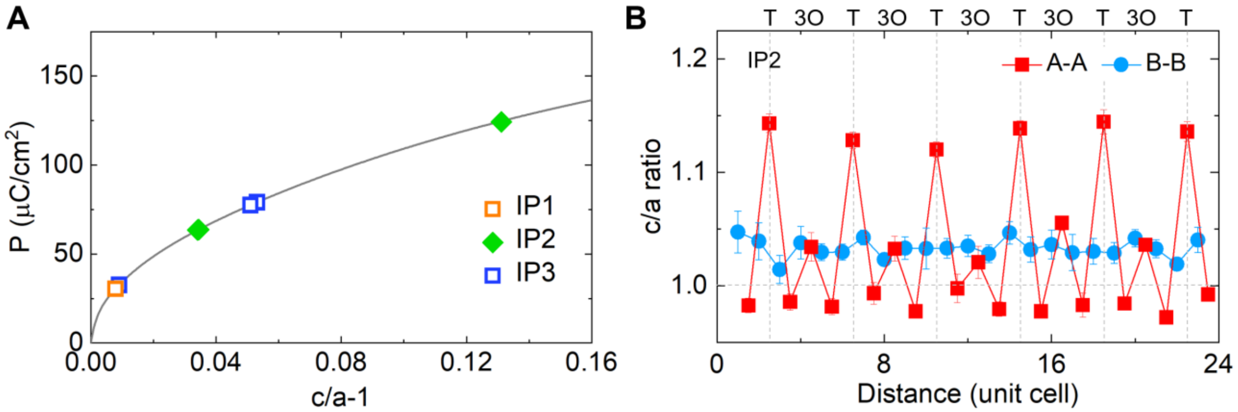

Fig. S5. Polarization evaluation based on $P^2 = \kappa (c/a - 1)$ relation in ordinary ferroelectrics. (**A**) The estimated polarization for IP1, IP2 and IP3 samples with the c_p/a_p ratio values measured from X-ray diffraction and atomic-resolution STEM measurement. (**B**) The lattice tetragonality (c/a) ratio of adjacent pseudocubic unit cells in the BM'' phase. Although the polarization in unit cells with small c/a ratio (< 1.0), measured from A-A columns, cannot be evaluated, the c/a ratio measured from B-B columns delivers a constant value, which corresponds to $\langle Pz \rangle$ = 63.7 μC/cm^2. The coefficient κ is determined by using $PbTiO_3$ with c/a = 1.064 and P = 82 μC/cm^2.

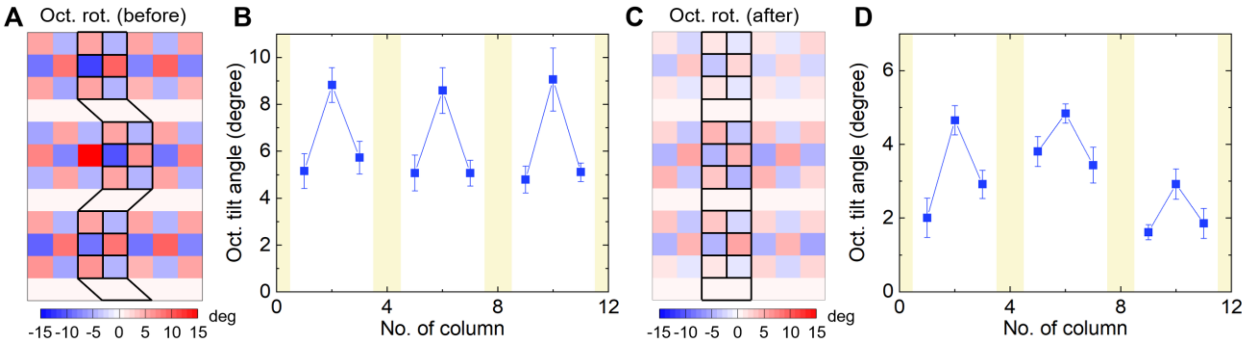

Fig. S6. Electron-beam irradiation induced collective octahedral rotation in the BM'' phase. (**A**,**C**) Anti-phase and in-phase octahedral rotation map of the BM'' phase before and after the electron-beam irradiation, respectively. (**B**,**D**) Plot of the octahedral rotation angle before and after the electron-beam irradiation, respectively.

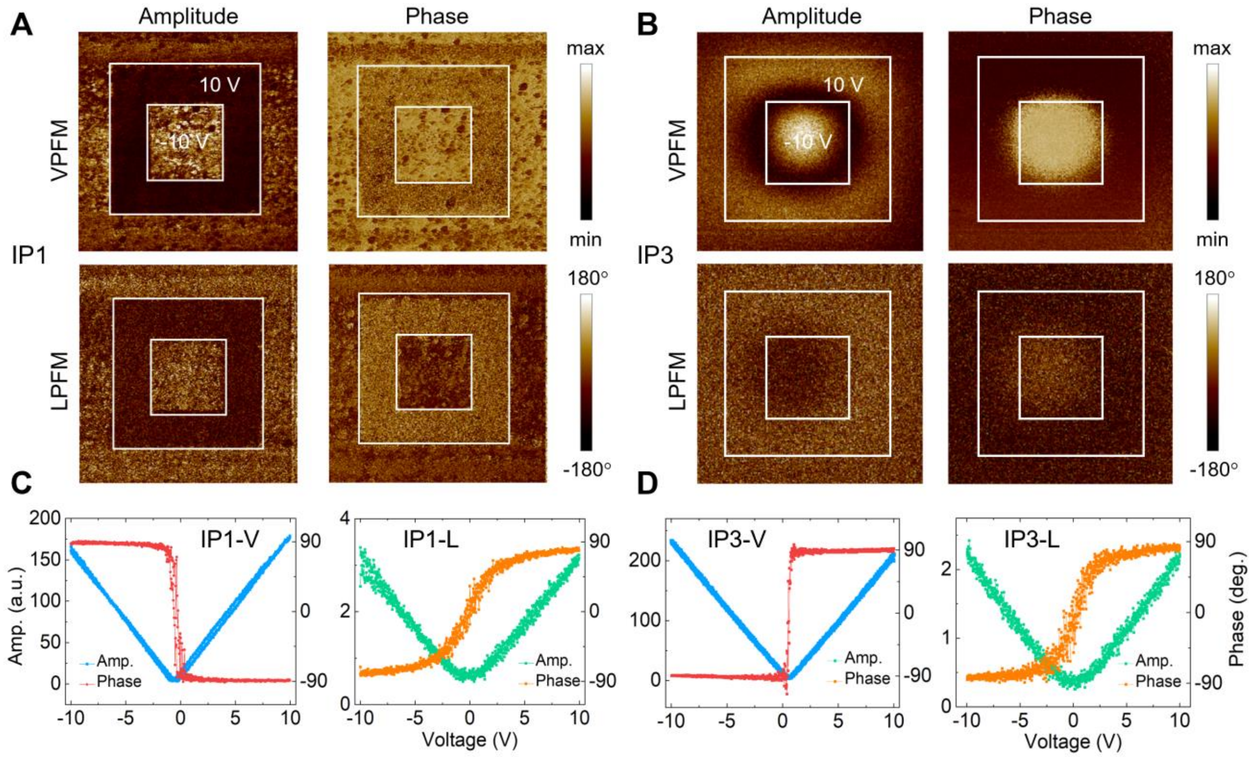

Fig. S7. Volatile piezo-response in the FD phase of NH-LSMO with δ ≈ 0.125 and 0.5. (**A**,**B**) Vertical (V) and lateral (L) PFM amplitude and phase maps of IP1 and IP3 samples with the marked regions poled at ± 10 V with a test frequency of f = 0.1 Hz and 0.2 Hz, respectively. (**C**,**D**) Typical V and L amplitude and phase hysteresis loops of the IP1 and IP3 samples, respectively. The size of each image is 15 × 15 μm^2.

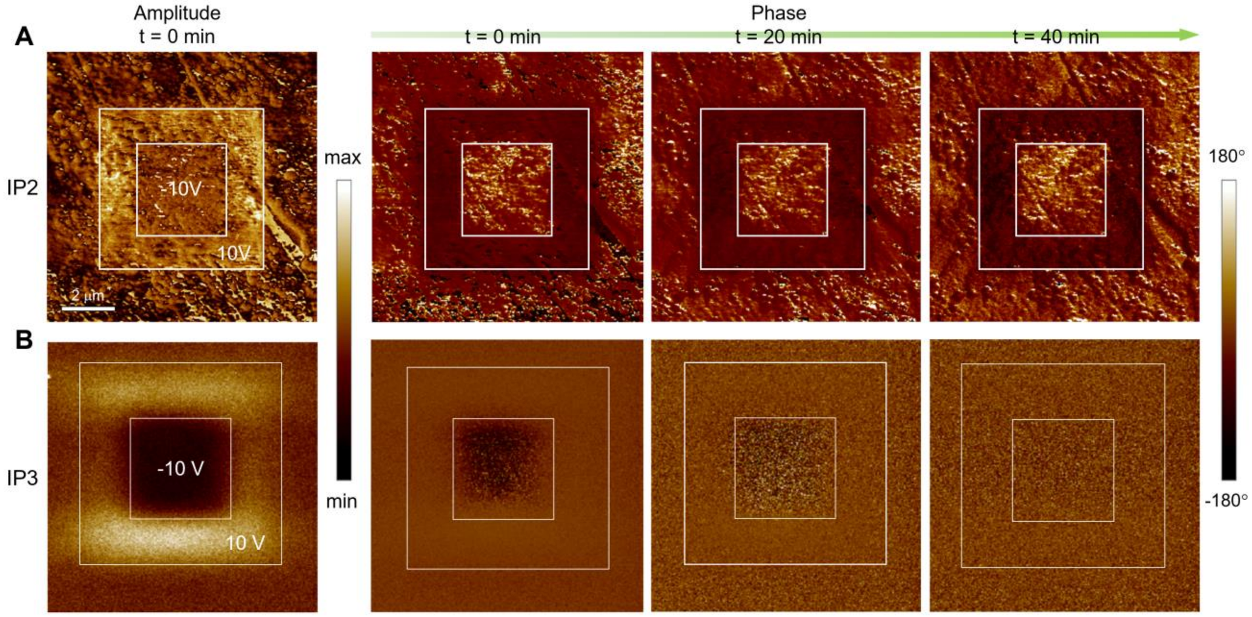

Fig. S8. Time dependent polarization retention in IP2 and IP3 samples. The square regions are vertically poled and switched by an applied PFM tip voltage of ± 10 V at t = 0 min and then re-scanned at t = 20 and 40 min, respectively.

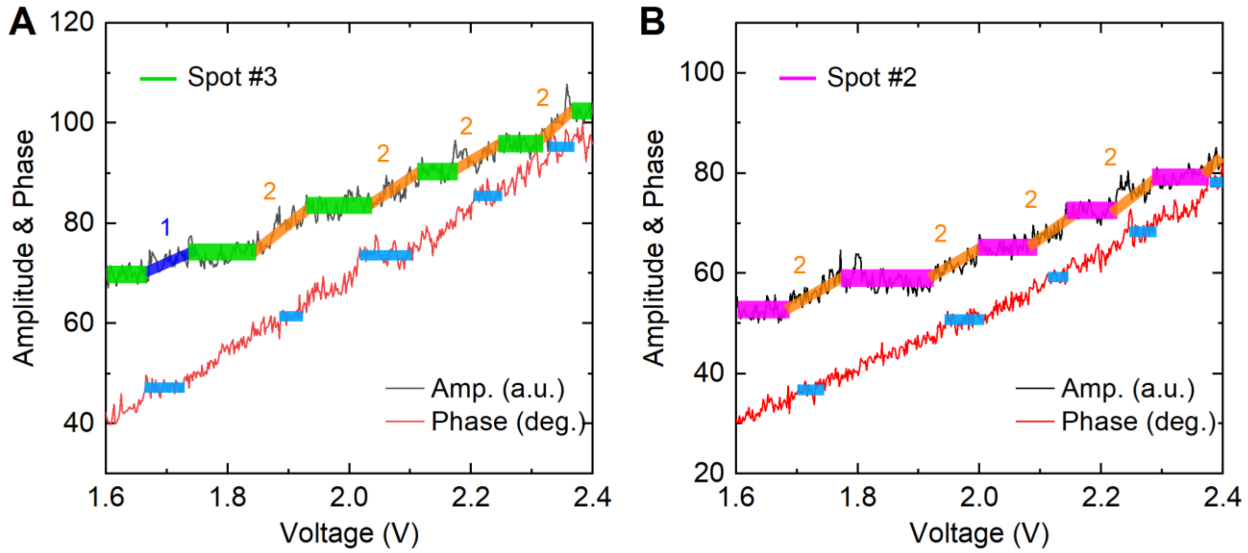

Fig. S9. Electric field-induced step-type PFM response. A,**B**, Voltage dependent amplitude and phase profiles for Spot #3 and #2 measured under application of a *dc* electric field, respectively.

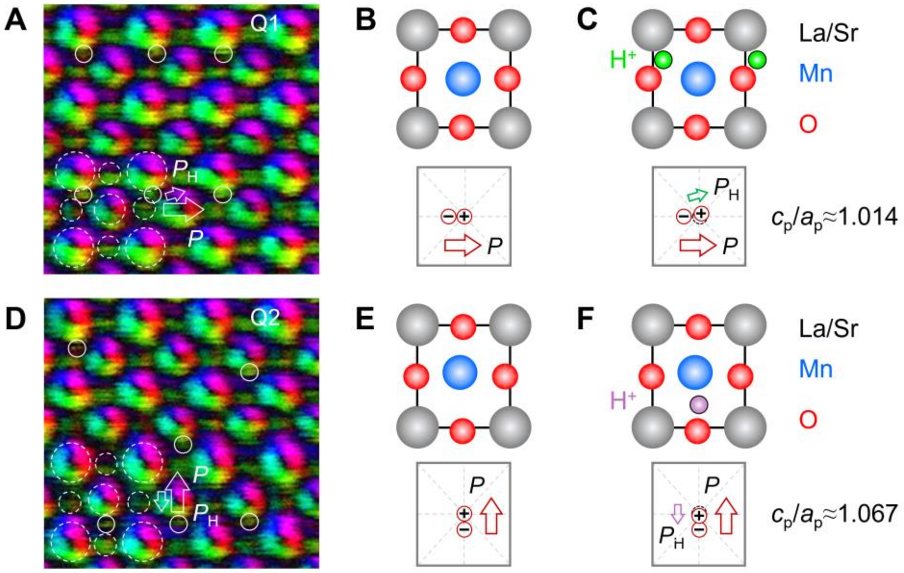

Fig. S10. H intercalation induced change in unit-cell polarization. (**A**,**D**) Magnified Q1 and Q2 regions with H atoms (solid circles) near the A-site (upper) and B-site (lower) columns, respectively. (**B**,**C**,**E**,**F**) The relative atom shifts without (**B**,**E**) and with (**C**,**F**) consideration of intercalated hydrogen in the form of protons. The H induced electric dipole is marked by P_H with respect to the H-free unit-cell dipole P.

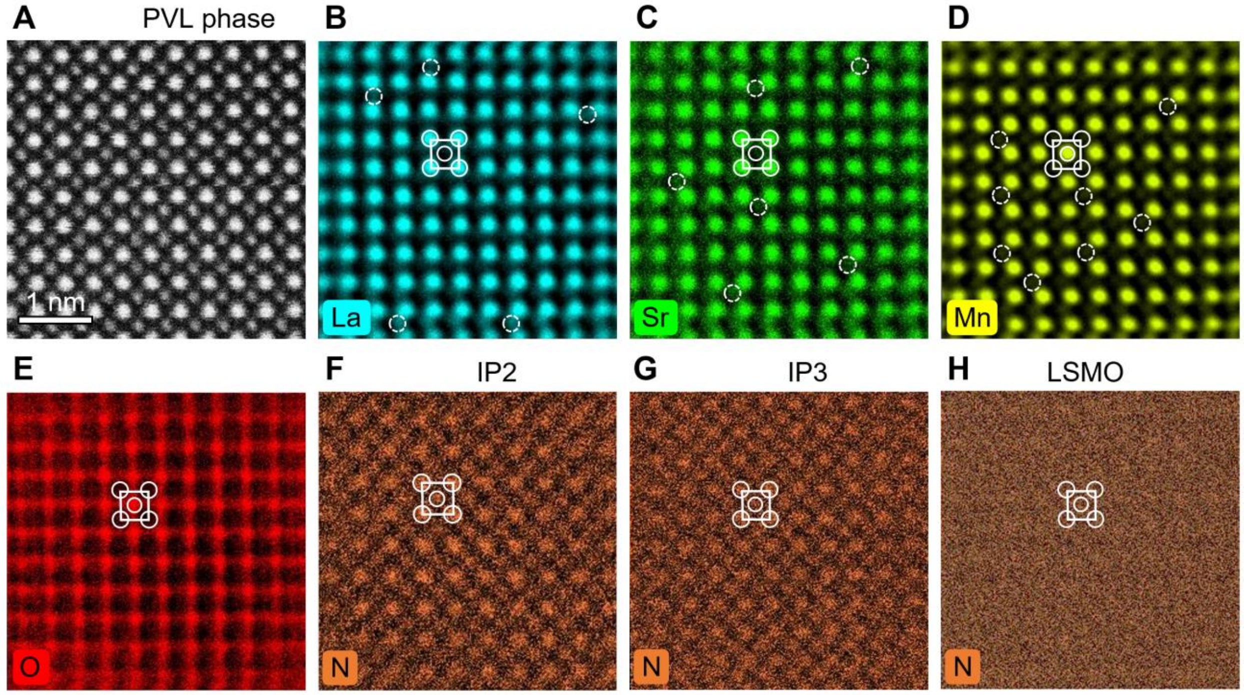

Fig. S11. Atomic-resolution EDS maps. (**A-E**) Representative elemental maps of La, Sr, Mn, O for the PVL phase, respectively. The white dashed circles mark the antisite defects such as La_O, Sr_{Mn}, Sr_O and Mn_A. (**F-H**) The EDS N maps for the IP2, IP3 samples and pure LSMO sample, respectively. The solid-line square denotes an La/Sr atom-based pseudocubic unit cell. One should note that during the long-time electron irradiation and data acquisition (~ 45 min for each set of data), the BM and BM'' phases have transformed to the PVL phase already.

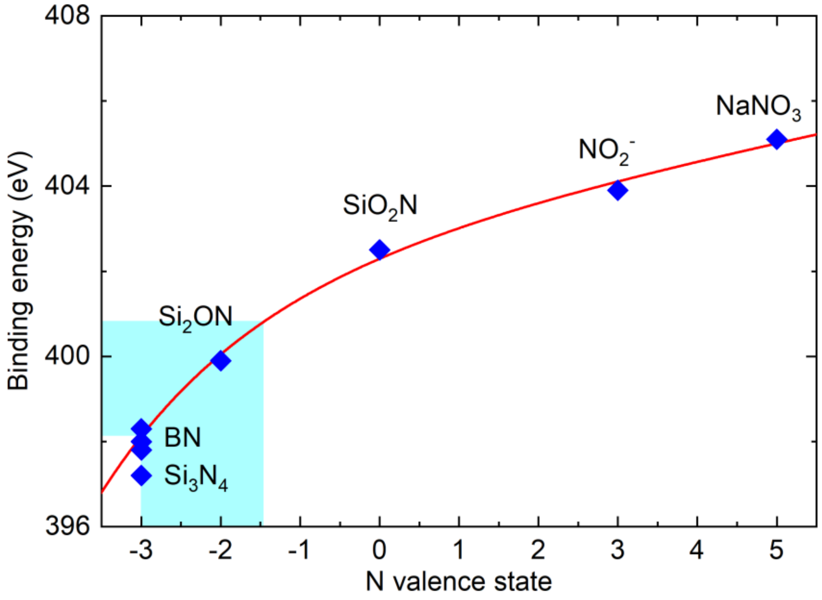

Fig. S12. Relationship of binding energy with N valence state in inorganic compounds. The names of specific compounds are labelled aside. The N valence state in the NH-LSMO samples are determined by referring to the fitted nonlinear profile (red solid line).
